# Life‐threatening peripartum cardiomyopathy—Not expected when expecting

**DOI:** 10.1002/ccr3.2158

**Published:** 2019-05-01

**Authors:** Peter Magnusson, Gabriella Kihlström, Marita Wallhagen, Komalsingh Rambaree

**Affiliations:** ^1^ Centre for Research and Development Uppsala University/Region Gävleborg Gävle Sweden; ^2^ Cardiology Research Unit, Department of Medicine Karolinska Institutet Stockholm Sweden; ^3^ Department of Building, Energy, and Environmental Engineering University of Gävle Gävle Sweden; ^4^ Department of Social Work and Psychology University of Gävle Gävle, Gävle Sweden

**Keywords:** implantable cardioverter defibrillator, left ventricular assist device, peripartum cardiomyopathy, sudden cardiac death, transplantation, wearable cardioverter defibrillator

## Abstract

Peripartum cardiomyopathy is challenging to diagnose as it mimics symptoms present in normal pregnancy. The clinical course and prognosis are various. In selected cases, a cardioverter implantable defibrillator with/without cardiac resynchronization therapy, mechanical ventricular assist device treatment, and transplantation is indicated.

## 1 INTRODUCTION

Peripartum cardiomyopathy (PPCM) is a rare cause of heart failure (HF) that affects women at the end of the pregnancy or in the months following delivery. The left ventricular ejection fraction (EF) is typically reduced below 45%. PPCM is a diagnosis of exclusion when no other cause of HF is found after extensive evaluation.^1^

Peripartum cardiomyopathy can affect women of all ethnicities in widespread geographical areas.^2^ However, there seems to be a predominance in certain areas as reported: one case per 102 deliveries in Zaira, Nigeria^3^ and 1:299 in Haiti^4^ while 1:571 in Sweden^5^ and 1:10 149 in Denmark.^6^ The etiology of PPCM is unclear and may be multifactorial.^1^ Angiogenic imbalance,^7^ genetic predisposition,^8^ altered prolactin processing,^9^ and myocarditis^10^ have been suggested as some of the possible causes.

A number of factors associated with increased risk of PPCM have been identified, for example, age above 30 years, pregnancy with multiple fetuses,^1^, ^11^ African descent,^12^ a history of preeclampsia, eclampsia, or postpartum hypertension.^13^ The majority of patients who develop PPCM do so during the first or second pregnancy.^13^

Symptoms of PPCM are dyspnea, decreased tolerance for physical exercise, persistent coughing, edema, chest discomfort, and palpitations consistent with other forms of HF. This may in part mimic limitations due to normal pregnancy.^1^ Differential diagnoses of PPCM are myocarditis, preexisting cardiomyopathy, valvular heart disease, congenital heart disease, myocardial infarction, pulmonary embolism, and amniotic liquid embolism.^14^

Some patients with PPCM recover from HF, but long‐term follow‐up studies are lacking. Occasionally, PPCM may be life‐threatening.^15^ We present a case of PPCM with an extremely complicated clinical course.

## 2 CASE PRESENTATION

A 27‐year‐old woman in gestational week of 40 + 2 was admitted to the hospital with chest discomfort, orthopnea, and sinus tachycardia 140 beats per minute (bpm) with QRS 96 ms.

She had a history of childhood focal glomerular sclerosis with nephrosis, which became steroid resistant and treated with chlorambucil, but recovered completely. Except for migraine occasionally, she was feeling healthy despite adiposity and body mass index 32 kg/m^2^ before pregnancy.

A computed tomography (CT) of the chest ruled out pulmonary embolism, but showed signs of edema enlargement of the left cardiac chambers; and echocardiography confirmed severe systolic dysfunction with left ventricular EF of 15%. The biomarker NT‐proBNP was elevated (1799 ng/L) but Troponin was normal. A suspicion of life‐threatening PPCM resulted in urgent air ambulance transport to the nearest university hospital, where caesarian section was promptly performed and a healthy child was delivered. At the intensive care unit, levosimendan was continued and standard HF initiated including furosemide, ramipril, metoprolol, aldosterone, digoxin, and warfarin. ECG showed premature ventricular complexes (PVCs). After 8 days, NT‐proBNP dropped to 881 ng/L.

Repeated echocardiography showed slight improvement; however, due to still deteriorated EF (28%), a wearable cardioverter defibrillator (WCD) or an implantable cardioverter defibrillator (ICD) were deemed unnecessary after extended discussions. In addition to low EF, secondary mitral insufficiency and elevated systolic right ventricular artery pressure (SPAP) developed in the patient.

In the following months, she suffered from HF in scale of New York Heart Association (NYHA) functional class III A‐B. The blood pressure (BP) was 90/60 mm Hg, and NT‐proBNP was increased to 813‐976 ng/L. Echocardiography confirmed an EF <30% and tricuspid annular plane systolic excursion (TAPSE) 1.6 cm.

In the meantime, the patient was repeatedly hospitalized for dizziness, dyspnea, and chest pain. After the sixth hospitalization, 3 months after onset of symptoms, she could not walk 50 m without limiting dyspnea; thus, she was referred to NYHA III or even IV. She was again transferred to the university hospital and, during the transportation, syncope spells occurred due to asystole. Based on the overwhelming risk of circulatory collapse, it was decided to start extracorporeal membrane oxygenation (ECMO). Echocardiography on the operating table showed stagnant left ventricle and the prognosis was considered extremely poor, and this was the reason that a left ventricular assist device (LVAD) HeartMate II™ was inserted. The insertion was complicated by critical ischemia in the right femoral communis artery, which was successfully treated with an embolectomy. Notably, even though the right ventricular function was moderately affected, the hemodynamic situation was stable after LVAD. Following HeartMate II™ initiation, she presented an infection, where an X‐ray later revealed tooth abscess but the dental procedure was postponed for 2 months because of instability; finally, eight teeth were extracted. Following HeartMate II™, her physical stamina improved (NYHA II), but syncopal spells occurred without detection of arrhythmia.

After HeartMate II™ insertion, she suffered from recurrent infection of the external driveline, with ultimate suspicion of biofilm around the cable. She was treated with long‐term antibiotics and symptoms improved. After 2 years with HeartMate II™, the decision was taken to initiate a transplant evaluation, and after another 6 months, she was placed on the waiting list.

During the period with HeartMate II™, the patient repeatedly experienced paroxysmal palpitations for less than a minute with dizziness, usually in the evenings and nights. Thumb‐ECG revealed monomorphic VT, and she was scheduled for a discussion about ICD. But at the following return visit, it appeared that the patient had hit her head and CT showed a small left occipital infarction. The contributing cause of the stroke was attributed to subtherapeutic international normalized ratio (INR). Two days later, a 24‐hour ambulatory ECG showed approximately 200 PVCs, 23 runs of nonsustained ventricular tachycardia (VT), at the most 20 beats in a row. Telemetry in the ward a few days later showed multiple broad complex tachycardias, likely of ventricular origin around 140 bpm. The patient received Mg^2+^, Na^+^, K^+^ drip, and amiodarone. Notably, she did not receive an ICD. She was advised to seek medical attention if the tachycardia was sustained for eventual cardioversion.

The patient experienced recurrent arrhythmias during the following years. One time at the emergency department, telemetry showed suspected polymorphic VT (*torsade de pointes*). It was speculated whether amiodarone caused proarrhythmia and was thus discontinued. A few months later, the patient suffered from repeated VTs followed by bradycardia with presyncope. Again, a discussion concerning ICD was initiated, but due to circulatory support of HeartMate II™, it was not implanted.

There were recurrent arrhythmias including atrial fibrillation, and the dose of beta‐blocker was increased. Notably, low levels of thyroid‐stimulating hormone with normal levels of thyroid hormones T4 (thyroxine) and T3 (triiodothyronine) and negative thyrotropin receptor antibodies were found. Thyroid scintigraphy revealed thyroiditis, assumed as a complication of amiodarone and was treated with glucocorticosteroids.

Due to adiposity and several treatment attempts by physiotherapists and dieticians, the body mass index ranges from 32 to 39 kg/m^2^ and the transplant team hesitated to offer her a transplant. However, 45 months after the onset of PPCM she finally received a new heart.

**Figure 1:**
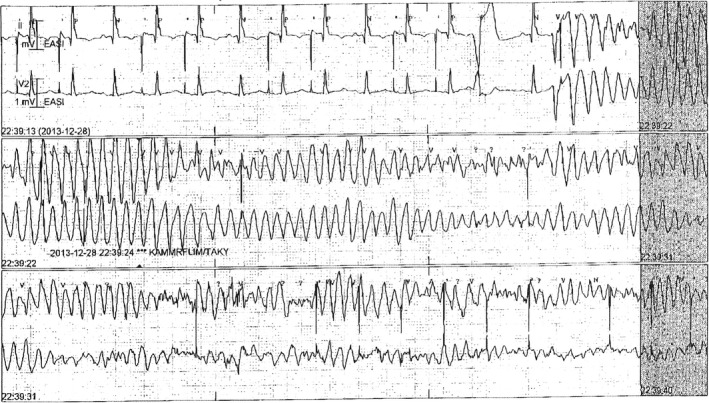
Ventricular fibrillation in a woman with peripartum cardiomyopathy

The transplant procedure was complicated due to the explant of HeartMate II™, and there was a weight mismatch between the donor and recipient. The subsequent HF required long‐term inotropic support. In the aftermath, she had renal failure with elevated creatinine (270 µmol/L). She also got another stroke in the right occipital lobe and suffered from visual defect. In addition, she had reactivation of cytomegalovirus infection and postoperative left‐sided diaphragm’s paralysis. Twelve days after heart transplantation, she had a witnessed cardiac arrest, with alternating QRS‐amplitude and axis, interpreted as torsade de pointes/ventricular fibrillation (Figure 1). Cardiopulmonary resuscitation (CPR) was performed for a few minutes including one successful defibrillation. Coronary angiography revealed no stenosis, and endomyocardial biopsy did not show any signs of rejection. After CPR, the patient developed an intense pain in the chest cavity resistant to treatment. There was no clear trigger for the cardiac arrest.

In the ward, there were runs of VTs after discontinuation of mexiletine and explant of a temporary pacemaker. Then she received an ICD (Current™ + DR). Echocardiography of the transplanted heart, performed during sinus rhythm of 65 bpm, showed EF 60%‐65%, TAPSE 6 mm, and SPAP 35 mm Hg. During the following year, the transplant resulted in rejection problems. At one time, combined cellular and humoral rejection occurred that was treated with high dose steroids and one dose of rituximab. Endomyocardial biopsy during the following years showed signs of mild to moderate rejection. Due to immunosuppression, she had pneumonia—four times during 18 months. After two strokes, the patient had sequelae in terms of some vision loss of the left eye. Unfortunately, she suffered from continued pain of different intensities since the PPCM diagnosis, accentuated by various causes such as the HeartMate II™, heart transplantation, and cardiac arrest with CPR and ICD implantation. During 2017, she experienced a lot of pain around the ICD pocket; therefore, it was decided to extract the ICD system 19 months after it has been implanted due to the severe pain problems and lack of need of pacing or antitachycardia pacing/cardioversion during the period, even though EF was 45%.

## 3 DISCUSSION

Diagnosing PPCM can be difficult since symptoms as nonspecific fatigue, shortness of breath, and pedal edema may mimic symptoms present in normal pregnancy.^1^ There is no specific ECG pattern for PPCM, but the ECG is rarely normal.^14^ Despite the severe form of PPCM, this patient had sinus tachycardia with otherwise normal ECG. Women presenting with PPCM typically have elevated plasma BNP or NT‐proBNP levels that are higher than seen in healthy women during pregnancy or postpartum.^16^ NT‐proBNP was markedly elevated. Echocardiography generally reveals a global reduction in left ventricular systolic function with EF <45% that is frequently, but not always, with dilatation.^1^ Echocardiography of this patient showed an EF of 15% at its worst. Moreover, cardiac magnetic resonance imaging is not mandatory for PPCM diagnosis, but in some cases it can rule out other differential diagnoses and improve assessment of morphology and function when echocardiography is inconclusive.^1^ In addition, it is noted that endomyocardial biopsy does not add any diagnostic or prognostic information in PPCM, but can be used to exclude acute myocarditis after delivery.^14^ Endomyocardial biopsy was performed around 3 months after the onset of symptoms and showed signs of chronic inflammation with increased levels of macrophages, lymphocytes, and actin filaments. PPCM is a diagnosis of exclusion, and in this case, pulmonary embolism was primarily suspected, which was reasonable because both pregnancy and the early postpartum period are associated with an increased risk of venous thrombosis and pulmonary embolism.^17^ However, the CT of the chest showed signs of edema and enlargement on the left cardiac side.

Treatment for women with PPCM who present after delivery should be treated according to the European Society of Cardiology guidelines for HF.^18^ Drugs used to treat chronic HF with reduced EF, including diuretic, beta‐blockers, nitrates, and digoxin, are recommended. Dopamine and levosimendan are administered intravenously and can be part of treatment in severe cases.^19^ During pregnancy, angiotensin‐converting enzyme (ACE) inhibitors, angiotensin II receptor blockers, and renin inhibitors are contraindicated because of fetal toxicity. After delivery, ACE inhibitors can be started. Mineralocorticoid receptor antagonists should be avoided during pregnancy and lactation. Furthermore, a diuretic should be administered with caution during pregnancy as it may impair perfusion of the placenta.^14^ During pregnancy, ivabradine is contraindicated because a shortage of evidence of safety during pregnancy; otherwise, it should be given even before or in parallel with beta‐blockers.^20^ In this connection, bromocriptine can be considered (2.5 mg twice daily for 2 weeks followed by 2.5 mg/d for 6 weeks).^21^ A German multicenter randomized trial with 63 women with PPCM with EF ≤35% treated with beta‐blockers, ACE inhibitors, and bromocriptine was associated with favorable outcomes.^21^ Additional treatments for patients with PPCM can be antiarrhythmic treatment, anticoagulation therapy, and mechanical support in selected cases.^1^

Anticoagulation with heparin should be started in all patients with acute PPCM treated with bromocriptine, because thromboembolic events have been reported as a complication, and those with severely reduced left ventricular systolic function (EF ≤ 35%).^14^ Accordingly, this patient did get bromocriptine for a couple of days after the caesarian section was performed in order to terminate lactation.

Arrhythmias are common in patients hospitalized for PPCM. A database study showed that 18.7% of patients who were hospitalized for PPCM had an arrhythmia (4.2% were VT and 2.2% cardiac arrest).^22^ Within this context, specific indications for the use of ICD therapy have not been established for PPCM.^1^ Decision‐making should therefore include a consideration of the natural history of these diseases, including the potential of recovery of ventricular function.^1^ In this sense, more studies with long‐term follow‐up of myocardial recovery are needed to describe the natural history of PPCM.^15^ Data on early recovery of left ventricular function are more frequently reported, and 20%‐60% of women with PPCM have complete recovery of EF within 6 months; but recovery can occur after 6 months, and continuing recovery can be seen after years.^15^ Mechanical circulatory support should be considered in patients with persistent hemodynamic instability with optimal pharmaceutical therapy.^14^ Cardiac resynchronization therapy, mechanical assist device treatment, and transplantation can become applicable in selected cases.^19^ In this particular case, the patient was given both ECMO and LV assist device.

Large randomized clinical trials showed that primary prevention use of an ICD improves survival in patients with cardiomyopathy and HF symptoms.^23^ A wearable cardioverter defibrillator (WCD) can be an alternative to prevent sudden cardiac death while waiting for decision to implant an ICD. For instance, an WCD may be indicated for patients with newly diagnosed nonischemic cardiomyopathy with severely reduced LV systolic function that is potentially reversible, and for patients with severe HF and patient who are awaiting heart transplantation.^24^, ^25^ The WCD appears equally efficacious among patients with and without myocardial ischemia prior to VT/ventricular fibrillation detection and shock.^26^ In this connection, prospective long‐term outcome data on large cohorts of patient with left LVAD and PPCM are lacking.^15^

In the Investigations of Pregnancy‐Associated Cardiomyopathy trial, there was cumulative event rate of 6% (death, heart transplantation, and LVAD) at 1 year in PPCM patients.^27^ Another study showed that up to 10% will require heart transplantation.^28^ A single‐center study showed that long‐term post‐transplant outcome for patient with PPCM was associated with favorable results.^27^

The role for ICD and WCD therapy in patients with LVAD is still unclear. One study has shown favorable survival with LVAD.^29^ It has not been studied whether WCD can provide similar survival benefits in patients waiting for transplantation with LVAD support.

In our case, which first happened 8 years ago, we would have advocated the use of WCD and subsequent ICD. There is a need for further larger, long‐term studies on prevention of sudden cardiac death and HF treatment in PPCM.

## 4 CONCLUSION

Peripartum cardiomyopathy may have an unpredictable and even life‐threatening clinical course. An optimal management including a multidisciplinary approach is warranted.

## CONFLICT OF INTEREST

None declared.

## AUTHOR CONTRIBUTION

PM: involved in idea, major writing of the article, and project management. GK: involved in extraction from medical records, drafting, and writing of the paper. MW and KR: involved in preparation and revision of the manuscript. All authors approved the final version of the case report for submission to the Clinical Case Reports.

## APPROVAL OF THE AUTHORS

All authors approved the final version of the case report for submission to the *Clinical Case Reports.*
